# An acute dose of inorganic dietary nitrate does not improve high-intensity, intermittent exercise performance in temperate or hot and humid conditions

**DOI:** 10.1007/s00421-018-04063-9

**Published:** 2019-01-08

**Authors:** Kieran Smith, David J. Muggeridge, Chris Easton, Mark D. Ross

**Affiliations:** 1 School of Applied Sciences, Edinburgh Napier University, Sighthill Campus, Edinburgh, UK; 2 Active Health Exercise Laboratory, Division of Biomedical Sciences, Institute of Health Research and Innovation, University of the Highlands and Islands, Inverness, UK; 3 Institute of Cellular Medicine, Faculty of Medical Sciences, Newcastle University, Newcastle upon Tyne, UK; 4 Institute for Clinical Exercise and Health Science, University West of Scotland, Hamilton, UK

**Keywords:** Nitrate, Exercise, Heat, High-intensity, Beetroot juice, Heat, Humidity

## Abstract

**Purpose:**

Dietary nitrate (NO_3_^−^) has repeatedly been shown to improve endurance and intermittent, high-intensity events in temperate conditions. However, the ergogenic effects of dietary NO_3_^−^ on intermittent exercise performance in hot conditions have yet to be investigated.

**Methods:**

In a randomised, counterbalanced, double-blind crossover study, 12 recreationally trained males ingested a nitrate-rich beetroot juice shot (BRJ) (6.2 mmol NO_3_^−^) or a nitrate-depleted placebo (PLA) (< 0.004 mmol NO_3_^−^) 3 h prior to an intermittent sprint test (IST) in temperate (22 °C, 35% RH) and hot conditions (30 °C, 70% RH). The cycle ergometer IST consisted of twenty maximal 6 s sprints interspersed by 114 s of active recovery. Work done, power output, heart rate and RPE were measured throughout; tympanic temperature was measured prior to and upon completion.

**Results:**

There were no significant effects of supplement on sprint performance in either temperate or hot, humid conditions (*p* > 0.05). There was a reduced peak (BRJ: 659 ± 100W vs. PLA: 693 ± 139W; *p* = 0.056) and mean power (BRJ: 543 ± 29W vs. PLA: 575 ± 38W; *p* = 0.081) following BRJ compared to PLA in the hot and humid condition, but this was not statistically significant. There was no effect of supplement on total work done irrespective of environmental condition. However, ~ 75% of participants experienced performance decreases following BRJ in the hot and humid environment. No differences were observed between trials for tympanic temperature measured at the conclusion of the exercise trial.

**Conclusion:**

In conclusion, an acute dose of inorganic dietary NO_3_^−^ does not improve repeated-sprint performance in either temperate, or hot and humid conditions.

## Introduction

Nitric oxide (NO) is a gaseous signalling compound associated with a plethora of physiological effects including modulating contractile properties of skeletal muscle (Ferguson et al. 2013), mitochondrial efficiency (Clerc et al. 2007; Heinonen et al. 2011) and peripheral/cutaneous blood flow (Lundberg et al. 2008). Circulating NO in the blood is short-lived and rapidly oxidised to nitrite (NO_2_^−^) and nitrate (NO_3_^−^). NO_3_^−^ is also known to be stored within skeletal muscle (Piknova et al. 2015) and the skin. Collectively they may act as a reservoir to ensure NO bioactivity is available during conditions of low pO_2_ (Lundberg et al. 2008), such as during intense physical exercise.

Dietary NO_3_^−^ has been shown to be effective at increasing circulating plasma NO_2_^−^ and NO_3_^−^ that coincides with improvement in indices of performance during cycling time trials (TT) (Cermak et al. 2012a; Lansley et al. 2011; Muggeridge et al. 2014), supra-maximal intensity cycling (Aucouturier et al. 2015) and explosive running (Sandbakk et al. 2015). This has been attributed to a reduced ATP cost during muscular contractions (Bailey et al. 2010) and potentially reduced $\dot {V}$O_2_ for mitochondrial ATP resynthesis, although the latter has failed to be confirmed more recently (Whitfield et al. 2015). However, some studies show that inorganic dietary NO_3_^−^ has been ineffective at improving performance (Cuenca et al. 2018; Sandbakk et al. 2015; Cermak et al. 2012b), which could be attributed to altered oral microbiota important for the initial conversation of NO_3_^−^ to NO_2_^−^ (Burleigh et al. 2018), chronic vs. acute dosages (Vanhatalo et al. 2010; Boorsma et al. 2014) and the level of athlete investigated, with those towards elite showing less of an ergogenic aid of nitrate than less-trained individuals (Porcelli et al. 2015).

NO-mediated physiological signalling following NO_3_^−^ supplementation is potentiated as the O_2_ (Castello et al. 2006) and pH (Modin et al. 2001) tension declines, therefore, NO_3_^−^ should in theory be more effective in high-intensity exercise as it creates favourable physiological conditions for NO production (Richardson et al. 1995). Dietary NO_3_^−^ supplementation has been reported to elevate skeletal muscle O_2_ delivery (Ferguson et al. 2013) and enhance sarcoplasmic calcium handling in fast twitch type II muscle fibres (Hernandez et al. 2012) translating to increased force production (Coggan et al. 2015). As such, high-intensity physical activities are more likely to increase NO synthesis from stored NO_3_^−^ reservoirs, and thus, improve performance (Wylie et al. 2016).

Exercise in the heat poses a formidable challenge to the body’s ability to control its internal environment through heat gain from external temperatures and high rates of metabolic heat production (Maughan and Shirreffs 2004). Given that cutaneous vasodilation is critical for the maintenance of a stable core temperature (*T*_c_), the role of dietary NO_3_^−^ supplementation in the heat warrants investigation. Indeed, the effect of dietary NO_3_^−^ supplementation on exercise performance in heat has recently been investigated in one non-athletic population (Kuennen et al. 2015) and in three studies of well-trained cyclists (Kent et al. 2018a, b; McQuillan et al. 2018). Following a moderate dose of inorganic dietary NO_3_^−^ (8.3 mmol NO_3_^−^·d^− 1^) for 6 days, Kuennen et al. (2015) observed a reduced O_2_ cost of a 45 min loaded march in a hot and humid environment compared to a PLA. Interestingly, it was shown that dietary NO_3_^−^ supplementation increased subject’s *T*_c_, a finding that was later replicated during a 4 km cycling TT in hot conditions (McQuillan et al. 2018). This may be due to elevated gastrointestinal blood perfusion, which may enhance thermal transfer during exercise in the heat. Additionally, the improved workload of the skeletal muscles could cause a subsequent ‘overspill’ of metabolic heat. However, this has most recently been disputed, where dietary NO_3_^−^ regimens have not influenced cycling TT performance (Kent et al. 2018b) or thermoregulatory responses in young adults (Amano et al. 2018) and elite cyclists (Kent et al. 2018a).

The prospective notion that dietary NO_3_^−^ supplementation alters heat tolerance is yet to be fully understood, where its effect on intermittent, sporting performance in trained individuals is yet to be investigated in hot conditions. As such, this investigation aimed to investigate whether an acute dose of inorganic dietary NO_3_^−^ would elicit performance benefits in recreationally trained males during an intermittent high-intensity exercise cycling protocol in temperate as well as in hot and humid conditions, with a potential improvement in performance resulting from an enhanced tissue and skin perfusion, resulting in enhanced O_2_ delivery, and heat dissipation. It was hypothesised that high-intensity, intermittent performance (mean and peak power; total work done) in the heat would improve following dietary NO_3_^−^ supplementation compared to a placebo in both conditions.

## Materials and methods

### Participants

Twelve recreationally trained male university students (22 ± 4 years, 1.81 ± 0.06 cm, 80.43 ± 5.84 kg, 46.11 ± 6.42ml kg min^−1^) volunteered to participate in the study. All participants had a history of competing at a high standard of team sports and had been training ≥ 2 times per week for at least 1 year. Participants gave their written consent prior to participation and all risks and potential benefits were fully explained prior to. The procedures employed in this study and risks were accepted in adherence to Edinburgh Napier University’s ethical committee and conformed to the code of ethics of the Declaration of Helsinki.

### Experimental design

Participants reported to the laboratory on five separate occasions. During the first visit, participants performed a ramp incremental test for assessment of $\dot {V}$O_2_ peak (see “Assessment of peak oxygen uptake”). After 20 min of recovery, participants then performed 10 min of the intermittent sports test (IST) in temperate conditions for individual gear calibration for the subsequent incremental exercise tests using a magnetically braked cycle ergometer (Velotron Pro, RacerMate Inc, USA). The 10 min IST required participants to perform five 2 min blocks (114 s of active recovery cycling at 100W maintaining 60 rpm and 6 s maximal sprint). Participants were asked during this session if they felt they could replicate this intensity for the full 40 min IST, following their response amendments were made to their gearing for the active recovery and maximal effort bouts.

Following completion of the preliminary testing, participants were assigned in a randomised, counterbalanced, double-blind, crossover experimental design to receive either an acute dose of NO_3_^−^-rich beetroot juice shot (BRJ: 6.2 mmol NO_3_^−^) or a NO_3_^−^-depleted placebo (PLA: <0.004 mmol NO_3_^−^), which they would ingest 3 h prior to the IST in temperate (22 °C, 35% RH) and hot conditions (30 °C, 70% RH). This dose of BRJ has been shown previously to improve exercise performance if ingested 2.5–3 h prior to exercise (Thompson et al. 2014; Hoon et al. 2014; Lansley et al. 2011). Randomisation was performed using an online programme, blinded to the researchers. At least 4–7 days separated each IST allowing for optimal recovery and supplement washout for circulating plasma NO_3_^−^ ([NO_3_^−^]) and [NO_2_^−^] levels to return to baseline (Wylie et al. 2013).

Prior to participation, all participants were instructed to fill out a food-screening questionnaire, detailing how often they ate certain foods and in what portion size. Participants were also asked to record their food intake 24 h prior to testing and were instructed to try and replicate this before subsequent sessions. All participants were given information regarding what foods contain the highest amount of NO_3_^−^·g^−1^ and to avoid consuming in high doses for the duration of the testing period. Participants were instructed to arrive to the laboratory in a fully rested, hydrated state at least 3 h postprandial and were advised to avoid any strenuous activity in the 24 h preceding each testing sessions. Caffeine and alcohol were to be refrained from consumption 6 h and 24 h, respectively, before each laboratory visit. Participants were also asked to abstain from antibacterial mouthwash and chewing gum use around supplement ingestion and experimental trials as these products have been previously shown to blunt the reduction of NO_3_^−^ to NO_2_^−^ in the oral cavity (Govoni et al. 2008). Testing all took place at the same time of day (± 3 h).

### Assessment of peak oxygen uptake

A $\dot {V}$O_2_ peak test to volitional exhaustion was performed on a Velotron Pro (RacerMate Inc, USA) cycle ergometer using a breath-by-breath gas analyser (CPX Jaeger, Germany), which monitored $\dot {V}$O_2_, $\dot {V}$CO_2_, and respiratory exchange ratio (RER). Participants warmed up for 5 min, cycling at an initial power output of 60W at 60–80 rpm. Following the warm up, in 1-min increments, resistance was increased by 30W until participants could no longer complete the 1-min step at 60–80 rpm or when they felt they could go on no further. $\dot {V}$O_2_ peak was taken as the highest mean-value attained during the final 30 s of exercise. HR was monitored throughout (Polar RS400 Heart Rate Monitors, Polar, Finland).

### Intermittent sport test (IST)

The IST was based on a motion analysis study of international field hockey players (Spencer et al. 2004) and is an abstract of the protocol previously described by Bishop and Claudius (Bishop and Claudius 2005). The IST, like the familiarisation and $\dot {V}$O_2_ peak session was performed on a Velotron Pro (Racer Mate, USA) cycle ergometer. All IST sessions took place in an environmental chamber (Weiss Gallenkamp, UK) in both temperate (22 °C, 35% RH) and hot and humid conditions (30 °C, 70% RH). Mean and peak power, work done, HR, and RPE were recorded after every sprint of the IST. Fatigue index per sprint was determined as: (maximum power – minimum power)/maximum power. Participant tympanic temperature (*T*_TYMP_) was measured upon commencement and immediately upon completion of the IST using a thermometer placed in the cavity of the ear (Braun IRT 4520, Braun ThermoScan, Germany).

Before the onset of the IST, a standardised warm up was completed comprising of cycling for 5-min at 100W at 60 rpm followed by a 2 min practice block of the IST. The 40 min IST replicates the duration of ‘one half’ of a rugby or hockey match, which was broken down into twenty × 2 min blocks consisting of a maximal 6 s sprint followed by 114 s active recovery. Participants were able to drink water *ad libitum*. The fixed resistance during the active recovery and maximum effort sprints were individually determined during the familiarisation session.

### Supplementation

Participants were randomly allocated in a crossover manor to consume either NO_3_^−^-rich BRJ (6.2 mmol NO_3_^−^ per 70 ml; Beet it, James White Drinks Ltd, United Kingdom) or a nitrate-depleted PLA (< 0.004 mmol NO_3_^−^ per 70 ml; Beet it, James White Drinks Ltd) shot identical in appearance and taste, administered in a double-blind fashion. Participants consumed their supplements 3 h prior to either the IST. Three hours prior to testing was chosen as pharmacokinetic data suggest that [NO_2_^−^] will be at its peak after a single dose of BRJ (Webb et al. 2008).

### Statistical analysis

All data were assessed for normal distribution. Data that were not normally distributed were logarithmically transformed (Log10). Paired sample *T*-tests were performed to compare the mean values of HR, delta *T*_TYMP_, peak power, mean power and mean work done per sprint and total work done during the IST between supplements (BRJ vs. PLA). The effect of inorganic dietary NO_3_^−^ on work done, power output, HR, RPE over the duration of the IST were analysed by a two-way repeated measures analysis of variance (ANOVA; time/sprint × condition). Cohen’s effect size (*d*) was calculated and expressed as: small effect > 0.2; medium effect > 0.5; large effect > 0.8. Inferential statistical analysis was conducted using the software package IBM SPSS Statistics (IBM Corp, USA). Data are presented as mean ± standard deviation (SD) unless stated otherwise. Significance was set at alpha ≤ 0.05.

## Results

### Physiological and perceptual responses

Upon termination of the IST, there were no differences in *T*_TYMP_ between BRJ and PLA in both temperate (BRJ: 35.8 ± 0.8 °C vs. PLA: 35.9 ± 0.5 °C, *p* = 0.78) and in the heat (BRJ: 37.3 ± 0.6 °C vs. PLA: 37.2 ± 0.6 °C, *p* = 0.93). Similarly, the increase in *T*_TYMP_ following the IST was not different between supplements (temperate: ΔBRJ: 0.57 ± 1.1 °C vs ΔPLA: 0.68 ± 0.33 °C; *p* = 0.74; heat: ΔBRJ: 1.49 ± 0.61 °C vs ΔPLA: 1.38 ± 0.7 °C; *p* = 0.37). There were also no differences in HR or RPE between supplements during the IST temperate (HR-BRJ: 151 ± 14 bpm vs. PLA: 151 ± 12 bpm; *p* = 0.94; RPE-BRJ: 14 ± 1 vs. PLA: 14 ± 2, *p* = 0.99). and in hot, humid conditions (HR-BRJ: 152 ± 17 bpm vs. PLA: 152 ± 16 bpm; *p* = 0.41; RPE-BRJ: 14 ± 1 vs. PLA: 14 ± 1, *p* = 0.74).

### Intermittent exercise performance

**Fig. 1:**
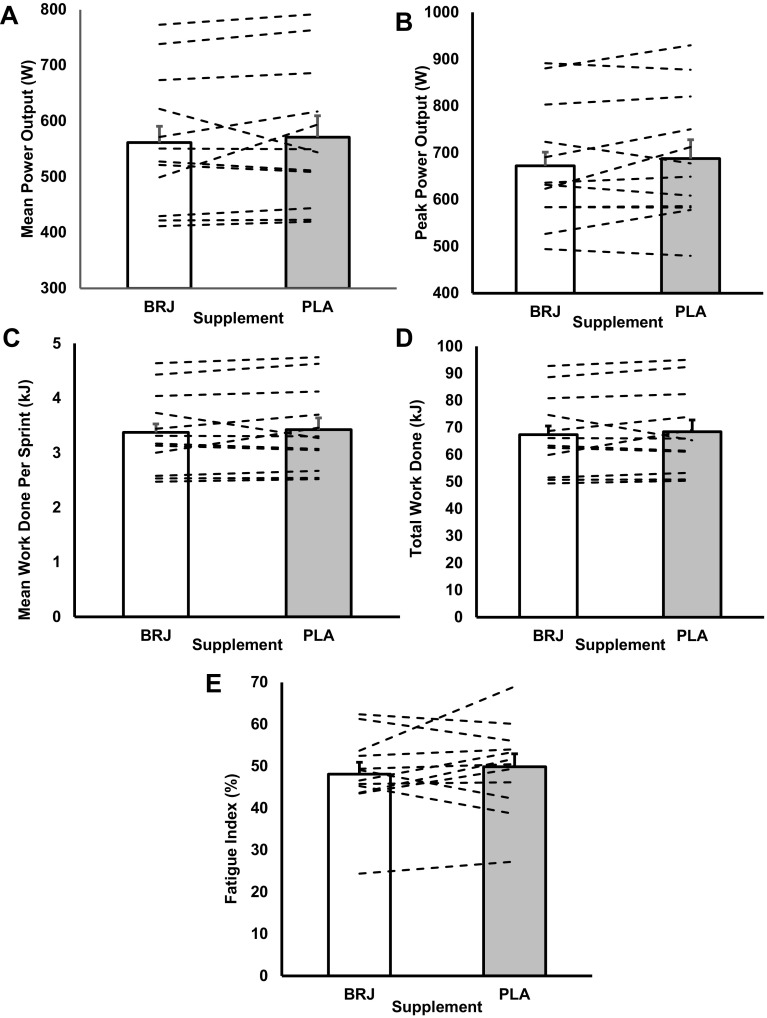
Mean peak power output (**a**), mean power production (**b**) and mean work done (**c**) produced per sprint during the intermittent sprint test (IST) in temperate conditions after ingesting either nitrate-rich beetroot juice (BRJ) or placebo (PLA). **d** Illustrates total work done across the twenty 6 s sprints during the IST and **e** represents fatigue index between trials. Dashed lines represent individual participant response. Data are presented as mean ± SEM

**Fig. 2:**
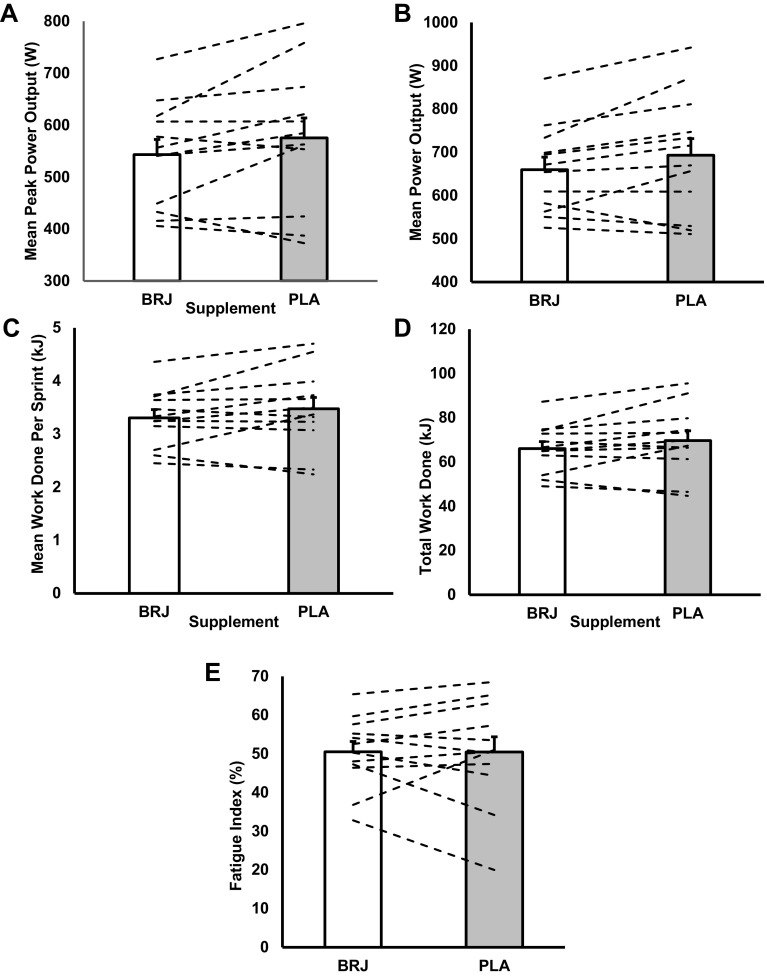
Mean peak power output (**a**), mean power production (**b**) and mean work done (**c**) produced per sprint during the intermittent sprint test (IST) in the heat after ingesting either nitrate-rich beetroot juice (BRJ) or placebo (PLA). **d** Illustrates total work done across the twenty 6 s sprints during the IST and **e** represents Fatigue Index between trials. Dashed lines represent individual participant response. Data are presented as mean ± SEM

There was no effect of dietary NO_3_^−^ ingestion on IST performance measures in temperate conditions (mean power production; BRJ: 562 ± 120W, PLA: 571 ± 124W, *p* = 0.433; total work done: BRJ: 67.44 ± 14.39 kJ, PLA: 68.46 ± 15.07 kJ, *p* = 0.447; Fig. 1). Mean power produced per sprint and total work done was reduced in BRJ than PLA in the heat, but these were not statistically significant differences (mean power production; BRJ: 543 ± 29W, PLA: 575 ± 39W, *p* = 0.081; total work done: BRJ: 66.07 ± 10.84 kJ, PLA: 69.74 ± 15.13 kJ, *p* = 0.101; Fig. 2). There was a trend for dietary NO_3_^−^ supplementation to reduce mean peak power production during the IST in the heat which neared statistical significance (*p* = 0.056; *d* = 0.28) compared to the PLA (Fig. 2). On average, peak power production in the heat was ~ 6% lower following BRJ (659 ± 100W) compared to PLA (683 ± 139W) (Fig. 2a, b).

**Fig. 3:**
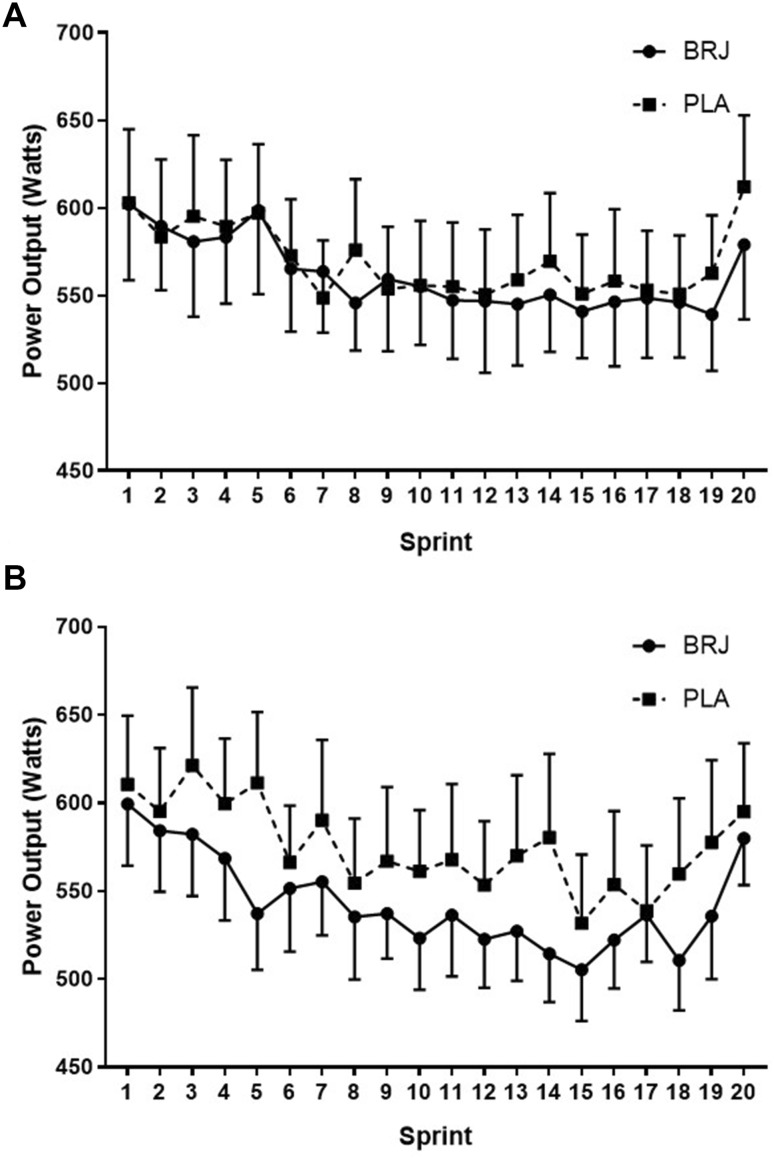
Mean power output during the intermittent sprint test (IST) following the nitrate-rich beetroot juice (BRJ; solid) and placebo (PLA; dashed) supplements in temperate (**a**) and hot and humid (**b**) conditions. Data are presented as mean ± SEM

There were no significant condition and sprint interactions for mean power production in both temperate (*F*_(19, 209)_ = 0.476, *p* = 0.971; Fig. 3a) and hot (*F*_(19, 209)_ = 1.147, *p* = 0.306; Fig. 3b) conditions. There was a trend for a lower mean power production per sprint following the BRJ (543 ± 29W) supplement compared to the PLA in the hot condition (575 ± 38W; *p* = 0.081; *d* = 0.34) (Fig. 2b).

**Fig. 4:**
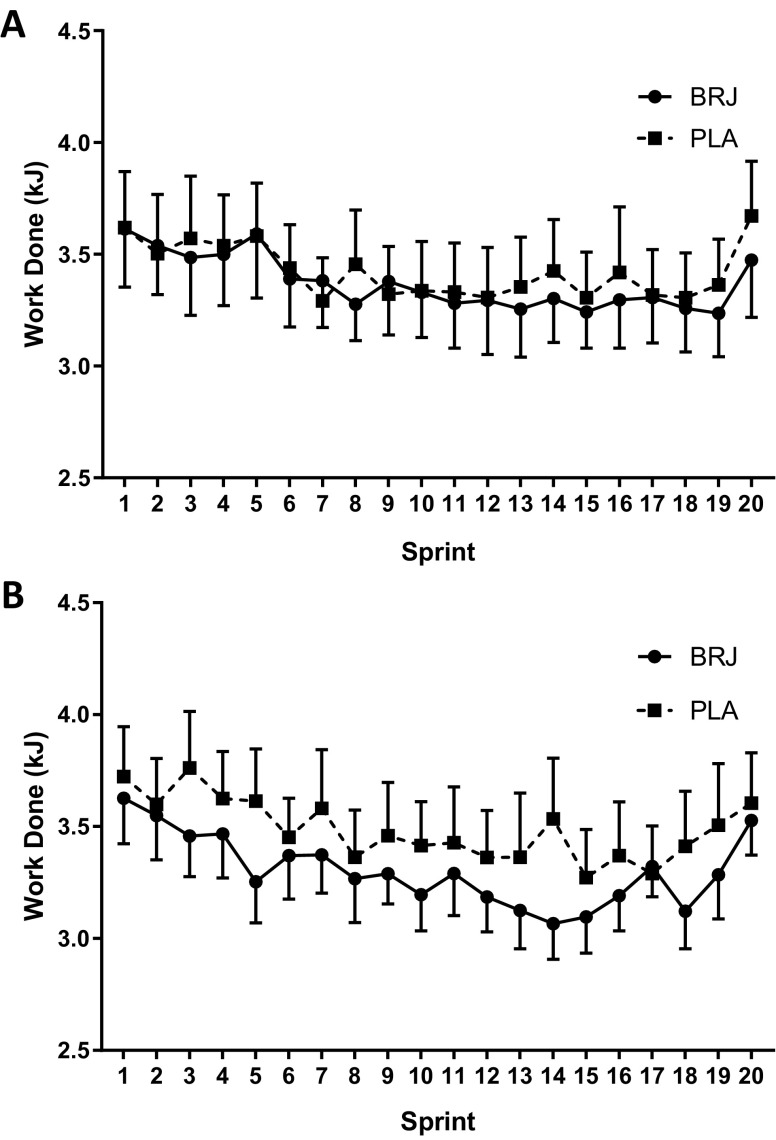
Mean Work Done per Sprint during the intermittent sprint test (IST) following the nitrate-rich beetroot juice (BRJ; solid) and placebo (PLA; dashed) supplements in temperate (**a**) and hot and humid (**b**) conditions. Data are presented as mean ± SEM

Likewise, no condition × sprint interaction effect was shown within mean work done for both temperate (*F*_(19, 209)_ = 0.498, *p* = 0.963; Fig. 4a) and hot conditions (*F*_(19, 209)_ = 1.062, *p* = 0.392; Fig. 4b). Mean work done per sprint was not different between supplements in either temperate (*p* = 0.45, *d* = 0.07; Fig. 1c) or hot conditions (*p* = 0.12, *d* = 0.26; Fig. 2c). BRJ did not influence total work done completed over the IST (temperate-BRJ: 67.44 ± 14.39 kJ vs. PLA: 68.46 ± 15.07 kJ) (*p* = 0.447, *d* = 0.07; Fig. 1d; hot-BRJ: 66.07 ± 10.84 kJ vs. PLA: 69.74 ± 15.13 kJ) (*p* = 0.101, *d* = 0.28; Fig. 2d). In addition, there was no difference in fatigue index between supplements (temperate-BRJ: 48.14 ± 9.77% vs. PLA: 49.89 ± 10.67%; *p* = 0.38 Fig. 1e; hot-BRJ: 50.51 ± 9.20% vs. PLA: 50.49 ± 13.51%; *p* = 0.99 Fig. 2e).

## Discussion

This is the first study to investigate the effect of dietary NO_3_^−^ supplementation on intermittent, high-intensity performance in both temperate as well as hot and humid conditions. Dietary NO_3_^−^ did not influence cardiovascular, perceptual or thermoregulatory responses to the exercise protocol, and appeared to impair some indices of performance, however this was only in the hot and humid condition, and was not statistically significant. This contrasts with previous research in temperate conditions which typically demonstrates that NO_3_^−^ is ergogenic for high-intensity intermittent exercise performance (Thompson et al. 2015, 2016; Wylie et al. 2013, 2016), with only one other study showing no effect of an acute NO_3_^−^ dose on intermittent exercise performance (Martin et al. 2014).

**Fig. 5:**
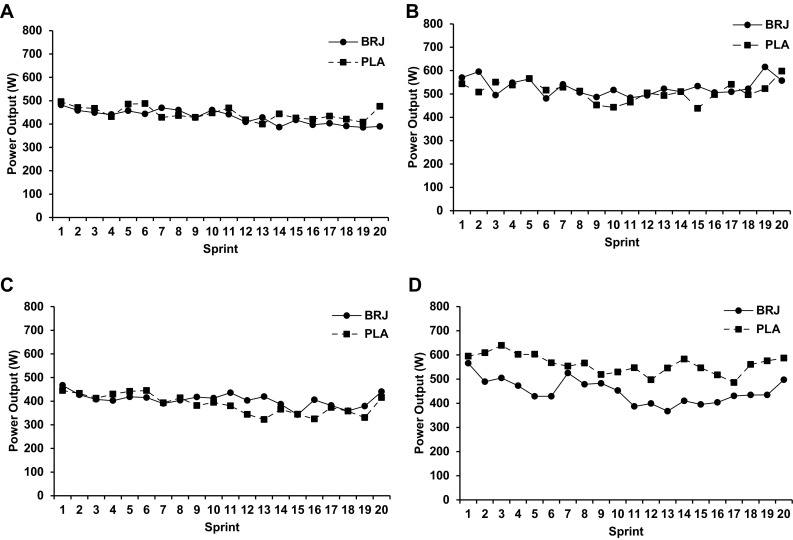
Mean Power Output during the intermittent sprint test (IST) following the nitrate-rich beetroot juice (BRJ; solid) and placebo (PLA; dashed) supplements in different participants. Example of one of four participants who showed little or no change in performance indices in the IST in temperate (**a**) and hot, humid conditions (**c**), and one example of the 8 eight participants who displayed decrements in performance in IST with BRJ supplementation (temperate: **b**, hot, humid: **d**)

The present investigation included 12 recreationally trained males, where following the ingestion of ~ 6 mmol NO_3_^−^, there was a trend for lower peak power (*p* = 0.056) and mean power production per sprint (*p* = 0.081) compared to the PLA trial in the hot condition only, with no such trend in temperate conditions. The reduction in power output with nitrate in the heat was observed in 8 out of the 12 participants, with the remaining four showing either no change, or slight improvement in power output (example figure provided; Fig. 5a–d). This is the first investigation to reveal such potential negative results following dietary NO_3_^−^ supplementation within a recreationally trained population, and appears to be only present in hot and humid conditions. In fact, lower doses of NO_3_^−^ (5–6 mmol NO_3_^−^) have produced favourable improvements in mean and peak power production during a 30 s Wingate test (Cuenca et al. 2018; Dominguez et al. 2017) and in cycling TT performances in both simulated altitude (Muggeridge et al. 2014) and normoxic conditions (Lansley et al. 2011). Speculatively, disparities between studies may be explained by the environmental conditions, where exercise in heat increases sympathetic nervous activity (Drust et al. 2005) influencing muscle metabolism (Febbraio et al. 1994) and vascular control (Johnson 2010).

Increases in muscle temperature can improve cross-bridge cycling rates (Karatzaferi et al. 2004) and sprint performance through enhancements in muscle fibre conductance (Girard et al. 2013; Gray et al. 2006). When recovery periods are long enough to allow for complete recovery between short duration sprints and in the absence of hyperthermia, there is little evidence to suggest hyperthermic conditions are detrimental to repeated-sprint performance compared to temperate conditions (Almudehki et al. 2012; Girard et al. 2013). Interestingly, we show that peak power production was lower following the BRJ supplement compared to PLA, nearing statistical significance (*p* = 0.056). Given type II muscle fibres are extensively recruited during shorter sprints compared to longer maximal efforts (Casey et al. 1996; Gray et al. 2008) and the known preferential NO_3_^−^-treatment fibre effects (Jones et al. 2016), such as preferential increases in blood flow to type II fibres (Ferguson et al. 2013), our findings are in stark contrast to previous literature within temperate environments (Thompson et al. 2015, 2016; Wylie et al. 2013, 2016), but the addition of a heat stress, as provided in our study, may compromise the ergogenic impact of inorganic NO_3_^−^ on performance.

It has been reported that dietary NO_3_^−^ supplementation augments an increase in *T*_c_ during exercise in the heat (Kuennen et al. 2015; McQuillan et al. 2018). The authors postulate that these effects may be specifically induced in metabolically active muscles, overriding the sympathetic vascular response in the skin that allows redistribution of blood flow to dissipate heat from the body (Crandall and Gonzalez-Alonso 2010). Whilst we displayed that *T*_TYMP_ rose to a similar extent in the BRJ and PLA conditions, it is plausible this may not fully represent the thermoregulatory responses our subjects may have experienced. Indeed, *T*_TYMP_ has been revealed to underestimate *T*_c_ during exercise in heat (Huggins et al. 2012). As such, *T*_TYMP_ measurements in this study may not accurately reflect any changes in *T*_c_ in our experiment. Increases in *T*_c_ during hyperthermic exercise creates a simultaneous demand for blood flow between active skeletal tissues, the skin and vital organs (Kent et al. 2018a); thus, influencing muscle metabolism and oxidative function (Febbraio et al. 1994), and subsequently limiting exercise performance (Drust et al. 2005).

Following local and whole-body heating, BRJ increases cutaneous vasodilation through NO-induced vasodilation despite not influencing skin blood flow suggesting no improved thermoregulatory benefit (Keen et al. 2015; Levitt et al. 2015). However, it has been reported that NO-dependent cutaneous vasodilation is diminished during high-intensity exercise in heat (Fujii et al. 2014). Given power output and total work done was lower in 19 out of the 20 sprints during the BRJ trial compared to the PLA in the heat (Figs. 3b, 4b), blood flow may have been preferentially distributed to other surrounding tissues or other neural thermoregulatory factors were at work (Drust et al. 2005; Febbraio et al. 1994). However, neither *T*_c_, *T*_sk_, peripheral nor muscle blood flow were measured in the present investigation leaving this open for future debate.

### Considerations

Larger or loaded dosages of inorganic dietary NO_3_^−^ have been consistently shown to improve repeated-sprint performance of short durations (Thompson et al. 2015; Wylie et al. 2013, 2016)—a hallmark of invasion team sports (Mohr et al. 2003; Spencer et al. 2004). However, we showed that the ingestion of dietary NO_3_^−^ 3 h prior to an IST in heat non-significantly reduced performance by 4–6%, which may represent a substantial performance detriment on the field of play. Despite this, our lack of benefit in the temperate conditions may be due to insufficient dose for this exercise mode, however, this dose did still correspond to a small reduction in performance indices in intermittent sprint activity in hot and humid conditions, as seen in this study. While these data seem highly relevant for competitive team sport athletes, they must be interpreted with caution. Our analysis was conducted on a small sample (*n* = 12) and differences between BRJ and PLA conditions were small and did not reach statistical significance, despite observing trends for impaired performance in the heat with BRJ supplementation. However, our findings of a potential negative impact of BRJ on performance in the heat, along with an absence of such negative impacts in ambient conditions, we can suggest that dietary NO_3_^−^ may impair high-intensity exercise performance in a recreationally trained population.

In addition, our measures of thermoregulation (*T*_TYMP_) are insufficient to fully understand the impact of nitrate on thermoregulation in hot and humid environmental conditions. Therefore future studies should employ more accurate measures of thermoregulatory strain, such as *T*_c_, *T*_sk_, sweat rate, muscle and skin blood flow. As a result of the small sample size, and the insufficient thermoregulatory measures, we are unable to specifically determine the physiological mechanisms that underpin this potential negative impact of BRF on high-intensity exercise in the heat.

## Conclusions

This study demonstrated that relative to the PLA, BRJ does not offer any beneficial aid to high-intensity, repeated-sprint performance in both ambient and hot conditions, but may be detrimental in the heat as demonstrated in our performance indices. However, with the trend of an acute dose potentially being ergolytic in hot and humid environments, the more common dietary NO_3_^−^ supplementary regimes of loading are postulated to be detrimental to repeated-sprint performance in heat through alterations in thermoregulatory responses and/or reductions in skeletal muscle blood flow. As such, we do not recommend athletes ingest dietary NO_3_^−^ supplements prior to high-intensity exercise in the heat.

## Abbreviations

BRJ: Beetroot juice
HR: Heart rate
IST: Intermittent sprint test
NO: Nitric oxide
NO_2_^−^: Nitrite
NO_3_^−^: Nitrate
PLA: Placebo
RER: Respiratory exchange ratio
RPE: Rating of perceived exertion
*T*_C_: Core temperature
*T*_sk_: Skin temperature
TT: Time trial
*T*_TYMP_: Tympanic temperature
